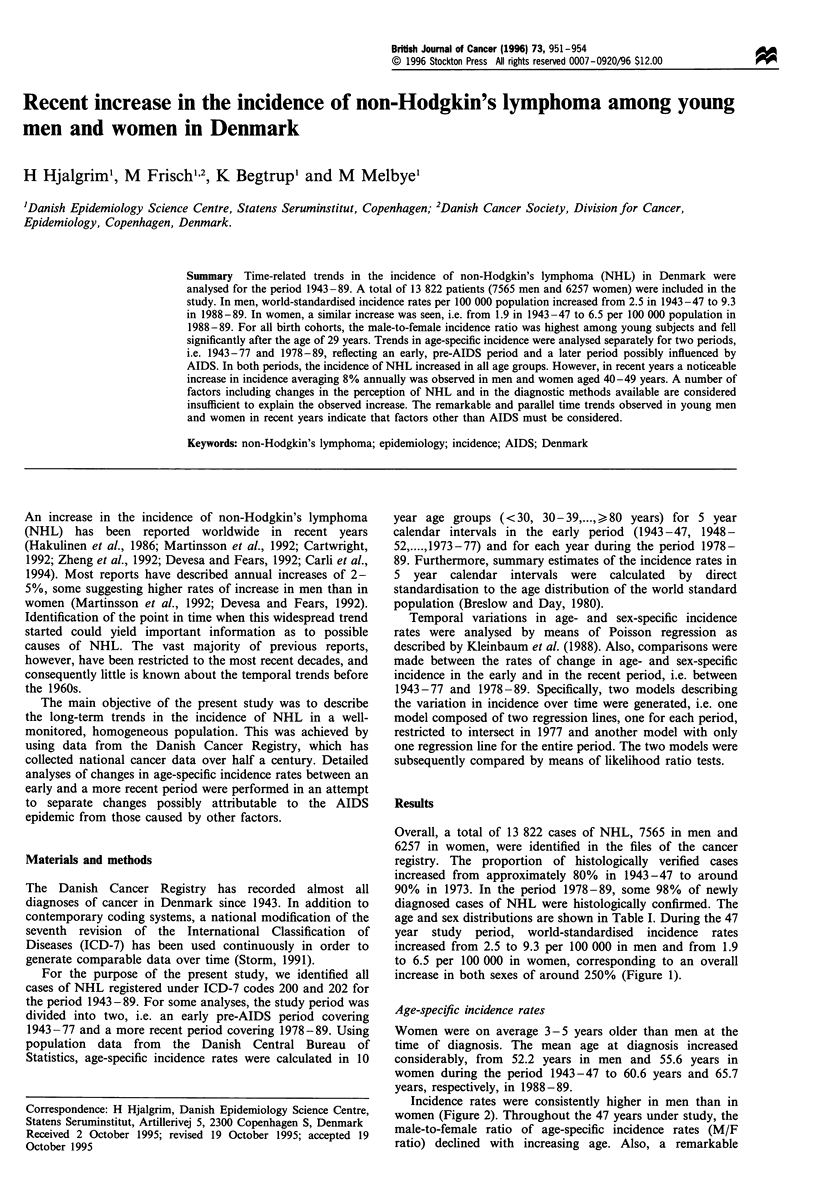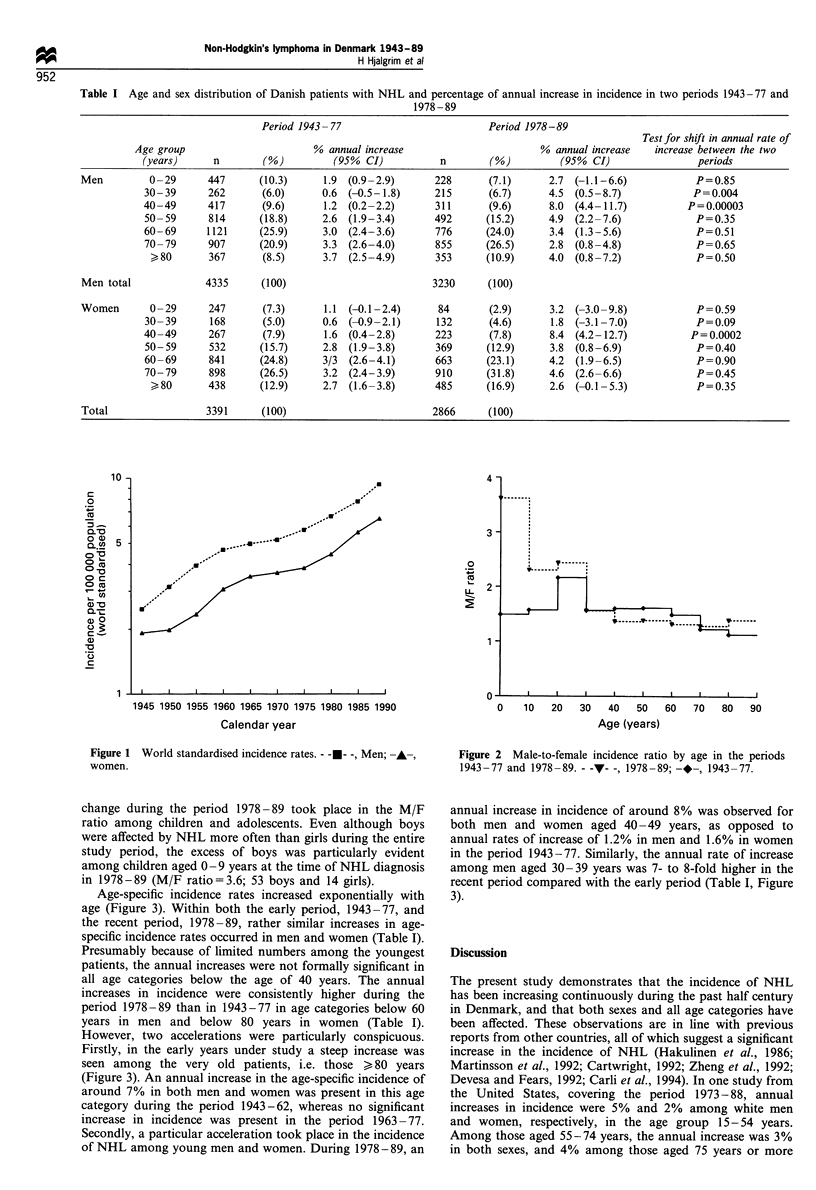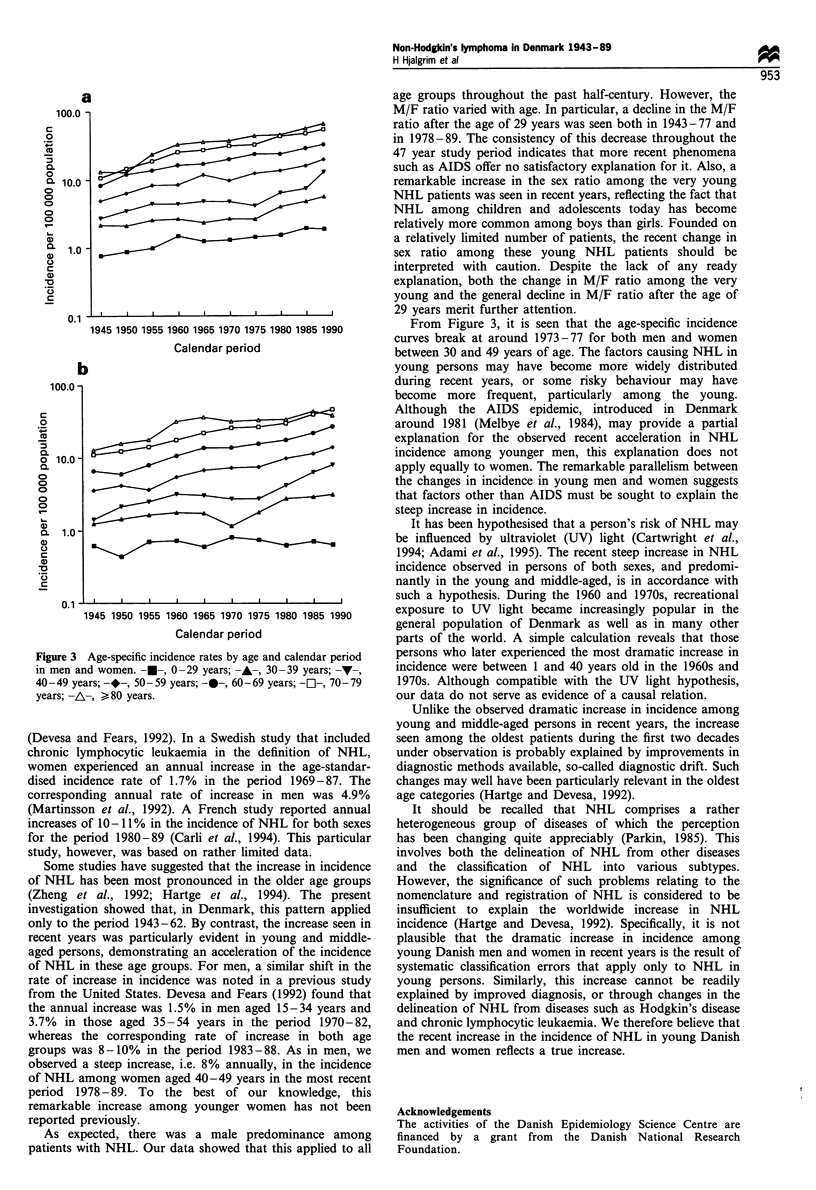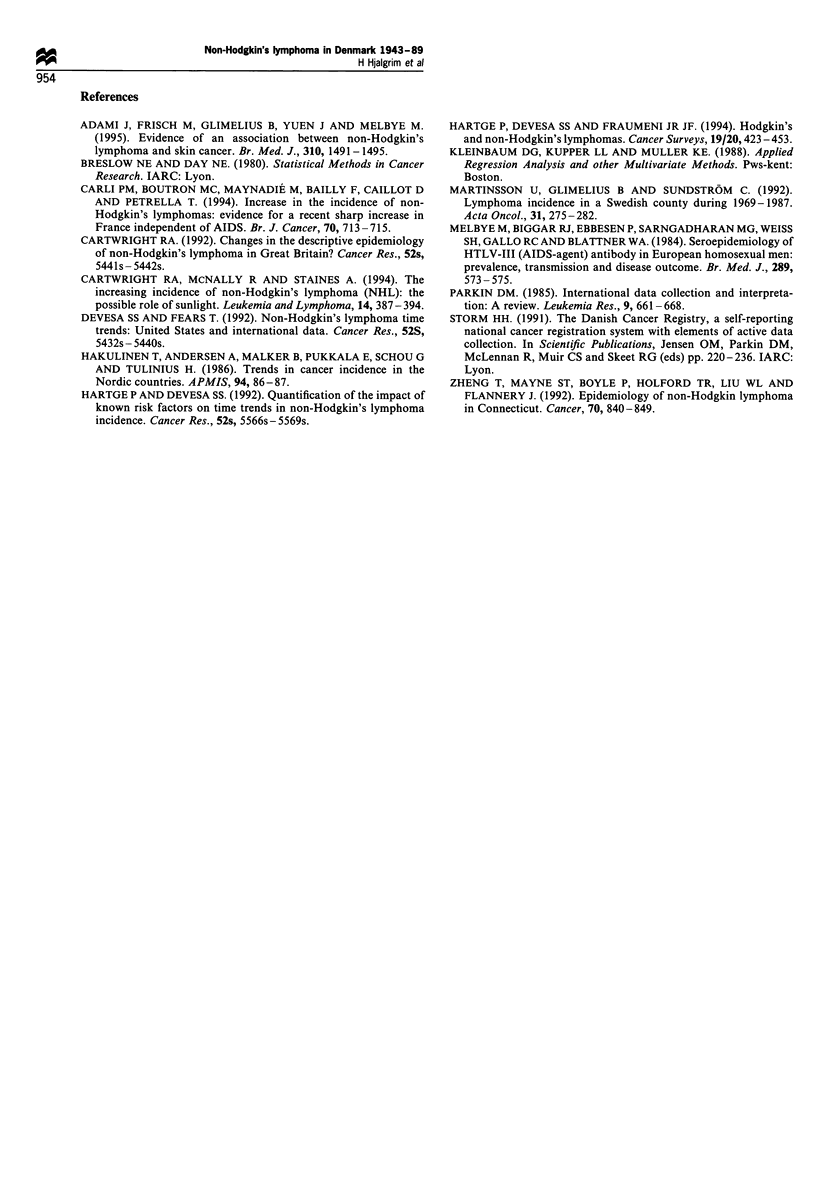# Recent increase in the incidence of non-Hodgkin's lymphoma among young men and women in Denmark.

**DOI:** 10.1038/bjc.1996.170

**Published:** 1996-04

**Authors:** H. Hjalgrim, M. Frisch, K. Begtrup, M. Melbye

**Affiliations:** Danish Epidemiology Science Centre, Statens Seruminstitut, Copenhagen, Denmark.

## Abstract

Time-related trends in the incidence of non-Hodgkin's lymphoma (NHL) in Denmark were analysed for the period 1943-89. A total of 13 822 patients (7565 men and 6257 women) were included in the study. In men, world-standardised incidence rates per 100 000 population increased from 2.5 in 1943-47 to 9.3 in 1988-89. In women, a similar increase was seen, i.e. from 1.9 in 1943-47 to 6.5 per 100 000 population in 1988-89. For all birth cohorts, the male-to-female incidence ratio was highest among young subjects and fell significantly after the age of 29 years. Trends in age-specific incidence were analysed separately for two periods, i.e. 1943-77 and 1978-89, reflecting an early, pre-AIDS period and a later period possibly influenced by AIDS. In both periods, the incidence of NHL increased in all age groups. However, in recent years a noticeable increase in incidence averaging 8% annually was observed in men and women aged 40-49 years. A number of factors including changes in the perception of NHL and in the diagnostic methods available are considered insufficient to explain the observed increase. The remarkable and parallel time trends observed in young men and women in recent years indicate that factors other than AIDS must be considered.


British Journal of Cancer (1996) 73, 951-954
© 1996 Stockton Press All rights reserved 0007-0920/96 $12.00

### Recent increase in the incidence of non-Hodgkin's lymphoma among young men and women in Denmark

H Hjalgrim[1], M Frisch[1,2], K Begtrup[1] and M Melbye[1]

[1]*Danish Epidemiology Science Centre, Statens Seruminstitut, Copenhagen;* [2]*Danish Cancer Society, Division for Cancer, Epidemiology, Copenhagen, Denmark.*

**Summary** Time-related trends in the incidence of non-Hodgkin's lymphoma (NHL) in Denmark were analysed for the period 1943-89. A total of 13 822 patients (7565 men and 6257 women) were included in the study. In men, world-standardised incidence rates per 100 000 population increased from 2.5 in 1943-47 to 9.3 in 1988-89. In women, a similar increase was seen, i.e. from 1.9 in 1943-47 to 6.5 per 100 000 population in 1988-89. For all birth cohorts, the male-to-female incidence ratio was highest among young subjects and fell significantly after the age of 29 years. Trends in age-specific incidence were analysed separately for two periods, i.e. 1943-77 and 1978-89, reflecting an early, pre-AIDS period and a later period possibly influenced by AIDS. In both periods, the incidence of NHL increased in all age groups. However, in recent years a noticeable increase in incidence averaging 8% annually was observed in men and women aged 40-49 years. A number of factors including changes in the perception of NHL and in the diagnostic methods available are considered insufficient to explain the observed increase. The remarkable and parallel time trends observed in young men and women in recent years indicate that factors other than AIDS must be considered.

**Keywords:** non-Hodgkin's lymphoma; epidemiology; incidence; AIDS; Denmark

An increase in the incidence of non-Hodgkin's lymphoma (NHL) has been reported worldwide in recent years (Hakulinen *et al.*, 1986; Martinsson *et al.*, 1992; Cartwright, 1992; Zheng *et al.*, 1992; Devesa and Fears, 1992; Carli *et al.*, 1994). Most reports have described annual increases of 2-5%, some suggesting higher rates of increase in men than in women (Martinsson *et al.*, 1992; Devesa and Fears, 1992). Identification of the point in time when this widespread trend started could yield important information as to possible causes of NHL. The vast majority of previous reports, however, have been restricted to the most recent decades, and consequently little is known about the temporal trends before the 1960s.

The main objective of the present study was to describe the long-term trends in the incidence of NHL in a well-monitored, homogeneous population. This was achieved by using data from the Danish Cancer Registry, which has collected national cancer data over half a century. Detailed analyses of changes in age-specific incidence rates between an early and a more recent period were performed in an attempt to separate changes possibly attributable to the AIDS epidemic from those caused by other factors.

### Materials and methods

The Danish Cancer Registry has recorded almost all diagnoses of cancer in Denmark since 1943. In addition to contemporary coding systems, a national modification of the seventh revision of the International Classification of Diseases (ICD-7) has been used continuously in order to generate comparable data over time (Storm, 1991).

For the purpose of the present study, we identified all cases of NHL registered under ICD-7 codes 200 and 202 for the period 1943-89. For some analyses, the study period was divided into two, i.e. an early pre-AIDS period covering 1943-77 and a more recent period covering 1978-89. Using population data from the Danish Central Bureau of Statistics, age-specific incidence rates were calculated in 10 year age groups (<30, 30-39,..., ⩾80 years) for 5 year calendar intervals in the early period (1943-47, 1948-52,....,1973-77) and for each year during the period 1978-89. Furthermore, summary estimates of the incidence rates in 5 year calendar intervals were calculated by direct standardisation to the age distribution of the world standard population (Breslow and Day, 1980).

Temporal variations in age- and sex-specific incidence rates were analysed by means of Poisson regression as described by Kleinbaum *et al.* (1988). Also, comparisons were made between the rates of change in age- and sex-specific incidence in the early and in the recent period, i.e. between 1943-77 and 1978-89. Specifically, two models describing the variation in incidence over time were generated, i.e. one model composed of two regression lines, one for each period, restricted to intersect in 1977 and another model with only one regression line for the entire period. The two models were subsequently compared by means of likelihood ratio tests.

### Results

Overall, a total of 13 822 cases of NHL, 7565 in men and 6257 in women, were identified in the files of the cancer registry. The proportion of histologically verified cases increased from approximately 80% in 1943-47 to around 90% in 1973. In the period 1978-89, some 98% of newly diagnosed cases of NHL were histologically confirmed. The age and sex distributions are shown in Table I. During the 47 year study period, world-standardised incidence rates increased from 2.5 to 9.3 per 100 000 in men and from 1.9 to 6.5 per 100 000 in women, corresponding to an overall increase in both sexes of around 250% (Figure 1).

### *Age-specific incidence rates*

Women were on average 3-5 years older than men at the time of diagnosis. The mean age at diagnosis increased considerably, from 52.2 years in men and 55.6 years in women during the period 1943-47 to 60.6 years and 65.7 years, respectively, in 1988-89.

Incidence rates were consistently higher in men than in women (Figure 2). Throughout the 47 years under study, the male-to-female ratio of age-specific incidence rates (M/F ratio) declined with increasing age. Also, a remarkable

Correspondence: H Hjalgrim, Danish Epidemiology Science Centre, Statens Seruminstitut, Artillerivej 5, 2300 Copenhagen S, Denmark
Received 2 October 1995; revised 19 October 1995; accepted 19 October 1995

**Table I** Age and sex distribution of Danish patients with NHL and percentage of annual increase in incidence in two periods 1943–77 and 1978–89

| | Age group (years) | *Period 1943–77* n | (%) | % annual increase (95% CI) | *Period 1978–89* n | (%) | % annual increase (95% CI) | Test for shift in annual rate of increase between the two periods |
|---|---|---|---|---|---|---|---|---|
| Men | 0–29 | 447 | (10.3) | 1.9 (0.9–2.9) | 228 | (7.1) | 2.7 (–1.1–6.6) | $P=0.85$ |
| | 30–39 | 262 | (6.0) | 0.6 (–0.5–1.8) | 215 | (6.7) | 4.5 (0.5–8.7) | $P=0.004$ |
| | 40–49 | 417 | (9.6) | 1.2 (0.2–2.2) | 311 | (9.6) | 8.0 (4.4–11.7) | $P=0.00003$ |
| | 50–59 | 814 | (18.8) | 2.6 (1.9–3.4) | 492 | (15.2) | 4.9 (2.2–7.6) | $P=0.35$ |
| | 60–69 | 1121 | (25.9) | 3.0 (2.4–3.6) | 776 | (24.0) | 3.4 (1.3–5.6) | $P=0.51$ |
| | 70–79 | 907 | (20.9) | 3.3 (2.6–4.0) | 855 | (26.5) | 2.8 (0.8–4.8) | $P=0.65$ |
| | ⩾80 | 367 | (8.5) | 3.7 (2.5–4.9) | 353 | (10.9) | 4.0 (0.8–7.2) | $P=0.50$ |
| Men total | | 4335 | (100) | | 3230 | (100) | | |
| Women | 0–29 | 247 | (7.3) | 1.1 (–0.1–2.4) | 84 | (2.9) | 3.2 (–3.0–9.8) | $P=0.59$ |
| | 30–39 | 168 | (5.0) | 0.6 (–0.9–2.1) | 132 | (4.6) | 1.8 (–3.1–7.0) | $P=0.09$ |
| | 40–49 | 267 | (7.9) | 1.6 (0.4–2.8) | 223 | (7.8) | 8.4 (4.2–12.7) | $P=0.0002$ |
| | 50–59 | 532 | (15.7) | 2.8 (1.9–3.8) | 369 | (12.9) | 3.8 (0.8–6.9) | $P=0.40$ |
| | 60–69 | 841 | (24.8) | 3/3 (2.6–4.1) | 663 | (23.1) | 4.2 (1.9–6.5) | $P=0.90$ |
| | 70–79 | 898 | (26.5) | 3.2 (2.4–3.9) | 910 | (31.8) | 4.6 (2.6–6.6) | $P=0.45$ |
| | ⩾80 | 438 | (12.9) | 2.7 (1.6–3.8) | 485 | (16.9) | 2.6 (–0.1–5.3) | $P=0.35$ |
| Total | | 3391 | (100) | | 2866 | (100) | | |

**Figure 1** World standardised incidence rates. - -■- -, Men; –▲–, women.

**Figure 2** Male-to-female incidence ratio by age in the periods 1943–77 and 1978–89. - -▼- -, 1978–89; –◆–, 1943–77.

change during the period 1978–89 took place in the M/F ratio among children and adolescents. Even although boys were affected by NHL more often than girls during the entire study period, the excess of boys was particularly evident among children aged 0–9 years at the time of NHL diagnosis in 1978–89 (M/F ratio = 3.6; 53 boys and 14 girls).

Age-specific incidence rates increased exponentially with age (Figure 3). Within both the early period, 1943–77, and the recent period, 1978–89, rather similar increases in age-specific incidence rates occurred in men and women (Table I). Presumably because of limited numbers among the youngest patients, the annual increases were not formally significant in all age categories below the age of 40 years. The annual increases in incidence were consistently higher during the period 1978–89 than in 1943–77 in age categories below 60 years in men and below 80 years in women (Table I). However, two accelerations were particularly conspicuous. Firstly, in the early years under study a steep increase was seen among the very old patients, i.e. those ⩾80 years (Figure 3). An annual increase in the age-specific incidence of around 7% in both men and women was present in this age category during the period 1943–62, whereas no significant increase in incidence was present in the period 1963–77. Secondly, a particular acceleration took place in the incidence of NHL among young men and women. During 1978–89, an annual increase in incidence of around 8% was observed for both men and women aged 40–49 years, as opposed to annual rates of increase of 1.2% in men and 1.6% in women in the period 1943–77. Similarly, the annual rate of increase among men aged 30–39 years was 7- to 8-fold higher in the recent period compared with the early period (Table I, Figure 3).

### Discussion

The present study demonstrates that the incidence of NHL has been increasing continuously during the past half century in Denmark, and that both sexes and all age categories have been affected. These observations are in line with previous reports from other countries, all of which suggest a significant increase in the incidence of NHL (Hakulinen *et al.*, 1986; Martinsson *et al.*, 1992; Cartwright, 1992; Zheng *et al.*, 1992; Devesa and Fears, 1992; Carli *et al.*, 1994). In one study from the United States, covering the period 1973–88, annual increases in incidence were 5% and 2% among white men and women, respectively, in the age group 15–54 years. Among those aged 55–74 years, the annual increase was 3% in both sexes, and 4% among those aged 75 years or more

**Figure 3** Age-specific incidence rates by age and calendar period in men and women. -■-, 0–29 years; -▲-, 30–39 years; -▼-, 40–49 years; -◆-, 50–59 years; -●-, 60–69 years; -□-, 70–79 years; -△-, ≥80 years.

(Devesa and Fears, 1992). In a Swedish study that included chronic lymphocytic leukaemia in the definition of NHL, women experienced an annual increase in the age-standardised incidence rate of 1.7% in the period 1969–87. The corresponding annual rate of increase in men was 4.9% (Martinsson *et al.*, 1992). A French study reported annual increases of 10–11% in the incidence of NHL for both sexes for the period 1980–89 (Carli *et al.*, 1994). This particular study, however, was based on rather limited data.

Some studies have suggested that the increase in incidence of NHL has been most pronounced in the older age groups (Zheng *et al.*, 1992; Hartge *et al.*, 1994). The present investigation showed that, in Denmark, this pattern applied only to the period 1943–62. By contrast, the increase seen in recent years was particularly evident in young and middle-aged persons, demonstrating an acceleration of the incidence of NHL in these age groups. For men, a similar shift in the rate of increase in incidence was noted in a previous study from the United States. Devesa and Fears (1992) found that the annual increase was 1.5% in men aged 15–34 years and 3.7% in those aged 35–54 years in the period 1970–82, whereas the corresponding rate of increase in both age groups was 8–10% in the period 1983–88. As in men, we observed a steep increase, i.e. 8% annually, in the incidence of NHL among women aged 40–49 years in the most recent period 1978–89. To the best of our knowledge, this remarkable increase among younger women has not been reported previously.

As expected, there was a male predominance among patients with NHL. Our data showed that this applied to all age groups throughout the past half-century. However, the M/F ratio varied with age. In particular, a decline in the M/F ratio after the age of 29 years was seen both in 1943–77 and in 1978–89. The consistency of this decrease throughout the 47 year study period indicates that more recent phenomena such as AIDS offer no satisfactory explanation for it. Also, a remarkable increase in the sex ratio among the very young NHL patients was seen in recent years, reflecting the fact that NHL among children and adolescents today has become relatively more common among boys than girls. Founded on a relatively limited number of patients, the recent change in sex ratio among these young NHL patients should be interpreted with caution. Despite the lack of any ready explanation, both the change in M/F ratio among the very young and the general decline in M/F ratio after the age of 29 years merit further attention.

From Figure 3, it is seen that the age-specific incidence curves break at around 1973–77 for both men and women between 30 and 49 years of age. The factors causing NHL in young persons may have become more widely distributed during recent years, or some risky behaviour may have become more frequent, particularly among the young. Although the AIDS epidemic, introduced in Denmark around 1981 (Melbye *et al.*, 1984), may provide a partial explanation for the observed recent acceleration in NHL incidence among younger men, this explanation does not apply equally to women. The remarkable parallelism between the changes in incidence in young men and women suggests that factors other than AIDS must be sought to explain the steep increase in incidence.

It has been hypothesised that a person's risk of NHL may be influenced by ultraviolet (UV) light (Cartwright *et al.*, 1994; Adami *et al.*, 1995). The recent steep increase in NHL incidence observed in persons of both sexes, and predominantly in the young and middle-aged, is in accordance with such a hypothesis. During the 1960 and 1970s, recreational exposure to UV light became increasingly popular in the general population of Denmark as well as in many other parts of the world. A simple calculation reveals that those persons who later experienced the most dramatic increase in incidence were between 1 and 40 years old in the 1960s and 1970s. Although compatible with the UV light hypothesis, our data do not serve as evidence of a causal relation.

Unlike the observed dramatic increase in incidence among young and middle-aged persons in recent years, the increase seen among the oldest patients during the first two decades under observation is probably explained by improvements in diagnostic methods available, so-called diagnostic drift. Such changes may well have been particularly relevant in the oldest age categories (Hartge and Devesa, 1992).

It should be recalled that NHL comprises a rather heterogeneous group of diseases of which the perception has been changing quite appreciably (Parkin, 1985). This involves both the delineation of NHL from other diseases and the classification of NHL into various subtypes. However, the significance of such problems relating to the nomenclature and registration of NHL is considered to be insufficient to explain the worldwide increase in NHL incidence (Hartge and Devesa, 1992). Specifically, it is not plausible that the dramatic increase in incidence among young Danish men and women in recent years is the result of systematic classification errors that apply only to NHL in young persons. Similarly, this increase cannot be readily explained by improved diagnosis, or through changes in the delineation of NHL from diseases such as Hodgkin's disease and chronic lymphocytic leukaemia. We therefore believe that the recent increase in the incidence of NHL in young Danish men and women reflects a true increase.

**Acknowledgements**
The activities of the Danish Epidemiology Science Centre are financed by a grant from the Danish National Research Foundation.

### References

ADAMI J, FRISCH M, GLIMELIUS B, YUEN J AND MELBYE M. (1995). Evidence of an association between non-Hodgkin's lymphoma and skin cancer. *Br. Med. J.*, **310**, 1491-1495.

BRESLOW NE AND DAY NE. (1980). *Statistical Methods in Cancer Research.* IARC: Lyon.

CARLI PM, BOUTRON MC, MAYNADIÉ M, BAILLY F, CAILLOT D AND PETRELLA T. (1994). Increase in the incidence of non-Hodgkin's lymphomas: evidence for a recent sharp increase in France independent of AIDS. *Br. J. Cancer*, **70**, 713-715.

CARTWRIGHT RA. (1992). Changes in the descriptive epidemiology of non-Hodgkin's lymphoma in Great Britain? *Cancer Res.*, **52s**, 5441s-5442s.

CARTWRIGHT RA, MCNALLY R AND STAINES A. (1994). The increasing incidence of non-Hodgkin's lymphoma (NHL): the possible role of sunlight. *Leukemia and Lymphoma*, **14**, 387-394.

DEVESA SS AND FEARS T. (1992). Non-Hodgkin's lymphoma time trends: United States and international data. *Cancer Res.*, **52S**, 5432s-5440s.

HAKULINEN T, ANDERSEN A, MALKER B, PUKKALA E, SCHOU G AND TULINIUS H. (1986). Trends in cancer incidence in the Nordic countries. *APMIS*, **94**, 86-87.

HARTGE P AND DEVESA SS. (1992). Quantification of the impact of known risk factors on time trends in non-Hodgkin's lymphoma incidence. *Cancer Res.*, **52s**, 5566s-5569s.

HARTGE P, DEVESA SS AND FRAUMENI JR JF. (1994). Hodgkin's and non-Hodgkin's lymphomas. *Cancer Surveys*, **19/20**, 423-453.

KLEINBAUM DG, KUPPER LL AND MULLER KE. (1988). *Applied Regression Analysis and other Multivariate Methods.* Pws-kent: Boston.

MARTINSSON U, GLIMELIUS B AND SUNDSTRÖM C. (1992). Lymphoma incidence in a Swedish county during 1969-1987. *Acta Oncol.*, **31**, 275-282.

MELBYE M, BIGGAR RJ, EBBESEN P, SARNGADHARAN MG, WEISS SH, GALLO RC AND BLATTNER WA. (1984). Seroepidemiology of HTLV-III (AIDS-agent) antibody in European homosexual men: prevalence, transmission and disease outcome. *Br. Med. J.*, **289**, 573-575.

PARKIN DM. (1985). International data collection and interpretation: A review. *Leukemia Res.*, **9**, 661-668.

STORM HH. (1991). The Danish Cancer Registry, a self-reporting national cancer registration system with elements of active data collection. In *Scientific Publications*, Jensen OM, Parkin DM, McLennan R, Muir CS and Skeet RG (eds) pp. 220-236. IARC: Lyon.

ZHENG T, MAYNE ST, BOYLE P, HOLFORD TR, LIU WL AND FLANNERY J. (1992). Epidemiology of non-Hodgkin lymphoma in Connecticut. *Cancer*, **70**, 840-849.